# Can Beetroot (*Beta vulgaris*) Support Brain Health? A Perspective Review on Alzheimer’s Disease

**DOI:** 10.3390/nu17233790

**Published:** 2025-12-03

**Authors:** Rachel Kimble, Oliver M. Shannon

**Affiliations:** 1 Sport and Physical Activity Research Institute, University of the West of Scotland, Blantyre G72 0LH, UK; 2 Human Nutrition & Exercise Research Centre, Centre for Healthier Lives, Population Health Sciences Institute, Newcastle University, Newcastle upon Tyne NE1 7RU, UK; oliver.shannon@newcastle.ac.uk

**Keywords:** beetroot, nitrate, brain health, Alzheimer’s, phytochemicals

## Abstract

Alzheimer’s disease (AD), the leading cause of dementia, has limited treatment options despite extensive pharmacological research. This has increased interest in dietary strategies that act across multiple pathological mechanisms. Beetroot (*Beta vulgaris*), known for its cardiovascular and metabolic benefits, contains a distinctive combination of bioactive compounds including inorganic nitrate, betalains, and polyphenols. Together these constituents influence vascular function, oxidative stress, mitochondrial efficiency, inflammation, and the microbiota. Previous reviews have typically focused on dietary nitrate in dementia prevention or have examined nitrate and betalains separately. In contrast, this review synthesises evidence on beetroot as a combined neuroprotective food. Preclinical data indicate that beetroot and its key constituents enhance antioxidant defences, support neuronal bioenergetics, and modulate cholinergic and inflammatory pathways. Human studies further suggest that nitrate-rich beetroot can improve cerebral blood flow and vascular responsiveness, and that higher intakes of plant-derived nitrate are associated with reduced cognitive decline. However, findings are inconsistent, most trials are small and short in duration, and research directly involving people with AD is scarce. By integrating vascular, antioxidant, and microbiome perspectives, this review identifies beetroot as a promising yet underexplored dietary candidate for AD management. Further mechanistic studies and multidomain approaches combining metagenomics, biomarkers, neuroimaging, and cognitive outcomes are needed.

## 1. Introduction

Alzheimer’s disease (AD), the leading cause of dementia worldwide, imposes profound personal, social, and financial burdens, while creating major demands on healthcare infrastructures and long-term support services [1]. It is a progressive neurodegenerative disorder, characterised by the gradual loss of cognitive function, including memory decline, impaired thinking, and changes in behaviour. Despite advances in prevention research, an estimated 57 million people currently live with dementia, a figure expected to almost triple by 2050 in the absence of effective therapies [2]. Current pharmacological treatment options are limited. Cholinesterase inhibitors and glutamate receptor antagonists can provide modest symptomatic relief, are affordable, and are typically well tolerated [3]. However, they do not modify the underlying disease course. More recently developed monoclonal antibodies (e.g., Donanemab and Lecanemab) are formulated to modify the disease trajectory by removing amyloid-β (Aβ) plaques (discussed further below) from the brain and have shown modest cognitive and functional benefits [4,5]. Moreover, they are frequently accompanied by adverse effects and are not deemed to be cost-effective [6]. These limitations highlight the need for effective, scalable, well-tolerated and affordable approaches to prevention and management that complement existing therapies.

AD is understood as a multifactorial and progressive disorder beginning decades before symptoms. Many underlying mechanisms contribute to neurodegeneration, as reviewed in detail by Verma et al. [7]. Oxidative stress is a well-established driver of AD pathogenesis and progression [8,9]. Impaired antioxidant defence and redox disturbances promote lipid and protein oxidation, DNA injury, and inflammatory signalling, compromising neuronal function and structure [10]. Oxidative stress is therefore central to many other hallmark features of AD such as Aβ plaques, neurofibrillary tangles, mitochondrial dysfunction, and cholinergic insufficiency [11]. Additionally, progressive vascular dysfunction is implicated in AD. Impaired cerebral blood flow (CBF), endothelial injury, and blood–brain barrier breakdown can interact with amyloid, tau, and inflammatory processes to accelerate neurodegeneration [12]. Moreover, emerging evidence suggests that oral and gut microbiome dysbiosis may contribute to AD via systemic inflammation, microbial metabolites, blood–brain barrier disruption, and altered immune signalling [13,14]. The complexity in AD pathology helps explain why isolated nutrient interventions such as antioxidants have largely failed to alter disease trajectories [15].

**Figure 1:**
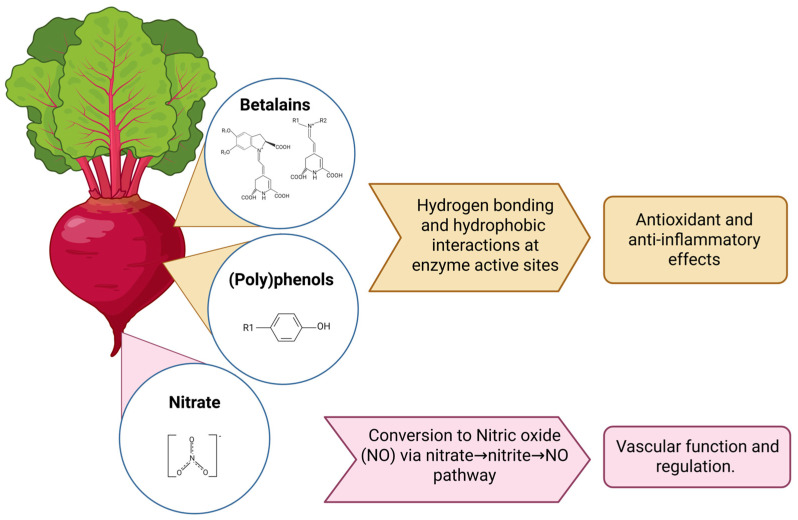
Beetroot phytochemicals reviewed and proposed mechanisms of action and key neuroprotective properties.

By contrast, dietary patterns and whole-food approaches are increasingly recognised for their ability to deliver a spectrum of bioactive compounds that act synergistically across multiple pathological pathways. Red beetroot (*Beta vulgaris* L.) is of particular interest in this context: it is naturally rich in inorganic nitrate (NO_3_^−^), betalains, (poly)phenols (Figure 1), and saponins and carotenoids, which will not be reviewed [16,17,18]. While the cardiovascular benefits of beetroot, mediated through nitric oxide (NO)-dependent vasodilation, are well documented, its potential to support brain health remains comparatively underexplored [19,20]. Earlier reviews have focused on dietary nitrate as a strategy to augment cognitive function in healthy individuals or to prevent dementia [21,22]. However, none have considered the other bioactive compounds and whether beetroot could be relevant for individuals already living with AD. Beetroot is inexpensive, widely accessible, and culturally adaptable, making it a potentially scalable dietary intervention for those with AD. Therefore, this review examines beetroot’s potential role in AD by synthesising evidence from mechanistic, epidemiological, and clinical research and aims to identify the key gaps that must be addressed.

## 2. Beetroot Neuroprotective Mechanisms

### 2.1. Nitrate and Brain Health

Beetroot is best known for its very high nitrate content (>250 mg/100 g fresh weight [23]), which provides a substrate for NO production via the enterosalivary nitrate–nitrite–NO pathway [24]. Leafy greens such as spinach, rocket, and lettuce along with celery and radish contain comparable or even higher nitrate concentrations, depending on their growing conditions. However, beetroot is widely studied in nitrate research due to its palatability, ease of consumption, and standardised, bioaccessible dose preparations [25]. Importantly, beetroot formulations (predominantly concentrated juices) have consistently been shown to increase NO bioavailability and improve cardiometabolic function [26,27,28]. This is noteworthy given the associations between vascular dysfunction and metabolic underpinnings in AD [29,30]. However, the role of nitrate and NO in the central nervous system remains complex, since excessive NO can be neurotoxic and may aggravate AD-related pathology [31].

**Figure 2:**
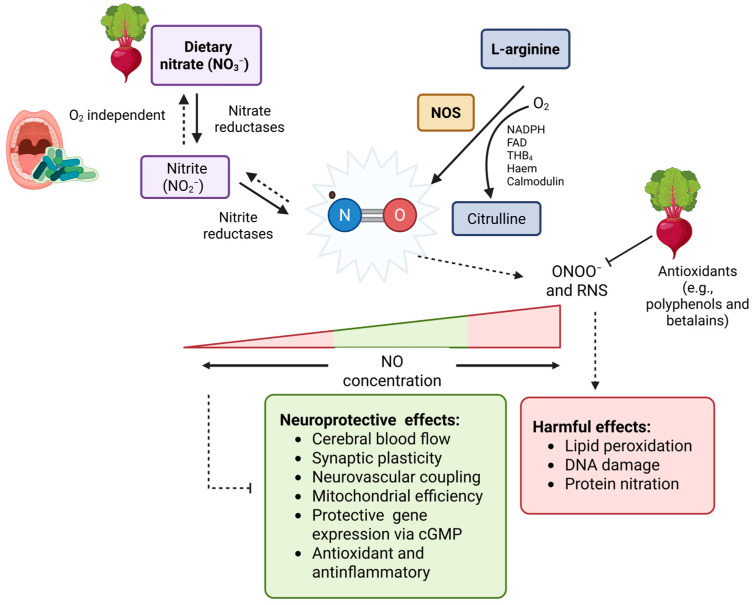
Nitric oxide (NO) is generated by nitric oxide synthases (NOS), which convert L-arginine and oxygen in the presence of essential cofactors [e.g., NADPH, flavin adenine dinucleotide (FAD), tetrahydrobiopterin (BH_4_), haem, and calmodulin], producing L-citrulline as a by-product. Alternatively, dietary nitrate, found in leafy greens and beetroot, can contribute to NO formation through its reduction to nitrite (NO_2_^−^) by oral bacteria (a rapid pathway) and xanthine oxidase (a slower pathway), which can be converted to NO via acidic reduction in the stomach or via various systemic nitrite reductases. This pathway is reversible hence the dashed arrows. The concentration of NO is relevant to its effects; while NO may be neuroprotective, high concentrations could contribute to oxidative and nitrosative stress when dysregulated. Although beetroots antioxidants may counteract this as discussed later.

NO is a pleiotropic signalling molecule with critical roles in vascular tone, synaptic plasticity, and neuroinflammation [32]. NO can be synthesised endogenously from the nitric oxide synthases (NOS) systems or from exogenous dietary sources such as beetroot [33] (Figure 2). Much of the evidence linking NO to AD pathology relates to the NOS system, where findings vary. In contrast, data on NOS-independent NO donors such as nitrate remain limited. Endogenous NO is produced from L-arginine by NOS, which exists in three major isoforms: endothelial (eNOS), neuronal (nNOS), and inducible (iNOS). The eNOS and nNOS are constitutively expressed and calcium-dependent, producing low, transient levels of NO, while iNOS is induced by inflammation and generates sustained, high concentrations [34]. Isoform-specific and context-dependent roles of NO are central to its duality; unlike NO produced by iNOS, NO derived from eNOS and nNOS seems to protect against AD neuropathology [35].

On the other hand, physiological levels of NO are essential for neuroprotection. NO supports neurovascular coupling, CBF, neuroplasticity, neurogenesis, and long-term potentiation (see [18,24]). However, classical NO-donating drugs have shown limited usefulness in AD because they have poor tissue distribution, short half-lives, and non-specific NO release, which can lead to systemic hypotension [36]. Nevertheless, several NOS−independent NO donors, including nitrates, are currently being prototyped for their therapeutic effects in AD (Table 1).

Dietary nitrate from beetroot also produces a slower and more sustained rise in NO that contributes modestly yet meaningfully to whole-body NO homeostasis [30]. Reduction to nitrite and subsequent NO could support brain health through established mechanisms including enhanced CBF and perfusion [41,42], antioxidant effects, and neuroprotection [43]. However, direct mechanistic evidence showing that dietary nitrate supplementation delivers sufficient NO to the brain is lacking. Nevertheless, there is some evidence for neuroprotective properties of whole beetroot interventions relevant to AD. For example, in a scopolamine-induced rat model of AD, Olasehinde et al. [44] supplemented the diet with 2 percent and 4 percent beetroot powder for 14 days. Both doses improved memory performance, with a better memory index in the 4%. These effects were linked to changes in purinergic enzyme activity, modulation of monoamine oxidase and angiotensin-converting enzyme activity and enhanced neuronal antioxidant status. In a separate study, red beetroot/carrot juice (80:20 *v*/*v*) given for 12 weeks attenuated cadmium-induced loss of calretinin-immunoreactive neurons [45]. These findings were attributed to the polyphenol content of the beetroot, although neither study measured nitrate, NO, or CBF directly.

### 2.2. Betalains and Polyphenols in Brain Health

Beetroot is also a rich source of phytochemicals such as betalains and polyphenols. Interest in these compounds stem from their antioxidant activities. In fact, beetroot ranks the highest among vegetables for total antioxidant capacity and contains the greatest phenolic content per dry weight [46].

Isolated beetroot compounds have been investigated for their in vitro and in vivo antioxidant effects. For example, 10, 20, and 50 μM of betanin increased cell viability in hydrogen peroxide-induced apoptosis in PC12 cells [47]. In the same study 5–50 μM of betanin was shown to reduce reactive oxygen species (ROS) production and decrease acetylcholinesterase (AChE) activity. In LPS-activated rat microglial cells, pretreatment with 500 μM of betanin was shown to reduce ROS levels, inflammatory cytokines [tumour necrosis factor-alpha (TNF-α), interleukin-6 (IL-6), and interleukin-1 beta), and cell injury [48]]. Together this was found with reduced mitochondrial membrane potential impairment and cellular energy status.

Similar findings have been reported in murine models. Shunan et al. [49] found that 10 and 20 mg/kg of betalains for 4 weeks suppressed aluminium-chloride-induced learning impairments in Sprague Dawley rats. In another study, 50 mg/kg of betanin attenuated induced memory and learning impairment in Scopolamine model of AD [50]. Both studies reported reductions in oxidative stress as well as improved histopathology. Moreover, Shunan and colleagues [49] found reduced AChE activity, while Salimi et al. [50] found improved mitochondrial function and structure.

The studies discussed above focused mainly on isolated phytochemicals such as betalains. However, limited work using whole beetroot interventions and computational docking approaches has shown that other beetroot polyphenols, including myricetin, catechin, quercetin, and apigenin, may bind to AD-related enzymes and alter their conformation and activity [44,51,52]. These findings highlight the need to study beetroot as a whole-food matrix, since the combined effects of its bioactive compounds may target multiple mechanisms relevant to AD.

### 2.3. Beetroot and the Microbiota–Brain Axis

In addition to their direct molecular effects, beetroot’s bioactive compounds may also act indirectly by shaping host–microbe interactions. The oral and gut microbiota, through the microbiota–brain axis, are now widely recognised as central mediators of diet–brain communication [53,54,55]. As such, examining these relationships may therefore offer valuable insight into how beetroot could contribute to cognitive health and the progression of AD. Nevertheless, only a limited number of studies have explored the potential for beetroot to modulate the microbiome [56,57,58].

In the oral cavity, commensal nitrate-reducing bacteria such as *Neisseria, Rothia*, and *Veillonella* convert dietary nitrate into nitrite, which is subsequently reduced to NO (Figure 2) [59]. Short-term beetroot juice intake has been shown to enrich nitrate-reducing bacterial modules (e.g., *Neisseria–Haemophilus*) while suppressing pathogenic species linked to cognitive decline and AD [60]. These microbial shifts correlated with improved cognitive performance in older adults, even in the absence of changes in CBF, suggesting beetroot may confer cognitive benefits through microbiome-mediated pathways. Oral dysbiosis is increasingly recognised as a modifiable risk factor for AD, with pathogens (e.g., *Porphyromonas gingivalis*) implicated in systemic inflammation, blood–brain barrier disruption, and direct neuroinflammatory activation [61]. By reducing the abundance of such pathogenic species while enriching nitrate-reducing commensals, beetroot may attenuate chronic low-grade inflammation and reduced NO bioavailability that may accelerate cognitive decline [62].

Within the gut, beetroot phytochemicals such as betalains and polyphenols reach the colon largely unmetabolised, where they undergo microbial transformation into bioactive metabolites with greater bioavailability and potential neuroprotective properties [63,64]. These compounds may exert prebiotic-like effects by shaping microbial composition and functional capacity. For example, in an eight-week intervention, daily consumption of 150 g of whole beetroot reduced the abundance of *Alistipes* in older adults [65]. This is a noteworthy finding given that this genus has been associated with impaired cognition [66] and AD pathology and may exacerbate AD through pro-inflammatory signalling [67]. The beetroot intervention increased production of short-chain fatty acids (SCFA), attributed to the higher fibre content of the whole beets [65]. Consistent with this, short-term beetroot juice supplementation has also been shown to elevate SCFA levels, with changes correlating to total excretion of betacyanins, further supporting a prebiotic role for beetroot and its phytochemicals [56]. SCFA are microbial metabolites capable of crossing the blood–brain barrier, modulating microglial activity, suppressing neuroinflammation, and attenuating Aβ and tau pathology [68], highlighting the potential relevance to AD.

## 3. Beetroot and Brain Health: Epidemiological Evidence and Clinical Insights

Although population-based studies have not specifically examined beetroot as a source of nitrate, emerging epidemiological evidence suggests that higher intakes of plant-based dietary nitrate may support brain health. In the Rush Memory and Aging Project, individuals with the highest nitrate intake experienced significantly slower rates of cognitive decline compared with those with the lowest intake [69]. By contrast, a cross-sectional analysis of NHANES data reported no consistent associations between urinary nitrate concentrations and cognitive performance, and even poorer Digit Symbol Substitution scores among participants with higher nitrate exposure [70]. Interpretation of these findings is limited by the use of a single spot urinary measure, which is influenced by hydration status, endogenous NO metabolism, and an inability to distinguish between nitrate from vegetables versus meats—sources that likely differ in predominance across populations.

More recent longitudinal evidence from the Australian Diabetes, Obesity, and Lifestyle study provides support for a neuroprotective role of plant-derived nitrate. In over 9000 participants, higher intakes of plant-based (~98 mg/day) and vegetable-based nitrate (~72 mg/day) were associated with 57% and 66% lower risks of dementia-related mortality, respectively [71]. Notably, higher intake of processed meat-derived nitrate was linked to a more than twofold increased risk of dementia mortality [HR (95% CI): 2.10 (1.07–4.12)], underscoring the importance of dietary source [72]. Complementary analyses in the Australian Imaging, Biomarkers and Lifestyle Study of Ageing demonstrated that among individuals at elevated genetic risk for AD, each additional 60 mg/day of plant-based nitrate at baseline was associated with better cognitive outcomes over 10.5 years, including higher episodic recall and recognition memory scores [73]. Extending this line of evidence, recent neuroimaging analyses by the same authors showed that in APOE ε4 carriers, moderate-to-high intakes of vegetable-sourced nitrate were linked to lower cerebral Aβ burden and attenuated right hippocampal atrophy [74].

Clinical studies have investigated dietary nitrate, primarily from concentrated beetroot juice, for its potential to augment CBF as a means to enhance cognitive function, though findings are mixed [75]. Beyond limitations such as small sample sizes and reliance on indirect measures of CBF, most trials have been conducted in young healthy adults (a population with already optimal CBF and cognitive function), thereby reducing the likelihood of detecting meaningful benefits. More promising results have emerged in older adults and those with vascular compromise. For example, Presley et al. [76] demonstrated that a high-nitrate diet supplemented with 16 oz beetroot juice (8.5 mmol nitrate) acutely increased regional perfusion in frontal white matter, particularly along tracts connecting the dorsolateral prefrontal cortex (DLPFC) and anterior cingulate cortex (ACC), despite no change in global perfusion. This is pertinent to AD, as arterial spin labelling studies consistently report hypoperfusion in the DLPFC and other frontal regions of AD patients compared with healthy controls [77]. Because the DLPFC–ACC network underpins executive control and is vulnerable to early AD-related vascular decline, enhanced perfusion in these tracts may represent a protective mechanism.

In patients with transient ischemic attack, 7-day sodium nitrate supplementation (0.1 mmol·kg^−1^·day^−1^) reduced blood pressure and cerebral artery velocity fluctuations while improving cerebral autoregulation [78]. Although this investigation did not involve beetroot directly, it reinforces the principle that nitrate supplementation can stabilise cerebrovascular dynamics even under pathological conditions. Clinical studies have also reported 2–12 weeks of beetroot juice intake reduces systemic oxidative stress and inflammatory markers [79,80,81,82]. Of particular note, a 12-week trial of concentrated beetroot juice (24 mL/day, 180 mg and ~46 mg of nitrate and polyphenols, respectively) in individuals with type 2 diabetes significantly decreased circulating IL-6 and TNF-α [80]. Given the established association between type 2 diabetes and increased AD risk [83], such reductions in systemic inflammation may be particularly relevant for modifying overlapping inflammatory pathways that contribute to neurodegeneration.

To date, however, no studies have directly assessed the effects of beetroot on CBF, cognitive function, or inflammation in AD populations. Notably, the only clinical evidence in people living with AD demonstrated that a single small dose of beetroot juice (5 mmol nitrate) increased systemic NO bioavailability (plasma nitrate and nitrite) to physiological levels comparable to healthy older adults and improved vascular responsiveness [84]. This finding is significant as it suggests that beetroot supplementation can augment vascular function even in the context of neurodegeneration and concomitant pharmacological treatment. However, there remain important gaps and limitations in the existing evidence that warrant careful consideration.

## 4. Current Gaps and Limitations

Current evidence for beetroot in AD is limited and remains fragmented across mechanistic, epidemiological, and clinical domains. Interpretation of the available data is further complicated by the dual nature of NO and methodological limitations in its measurement that do not distinguish enzymatic from non-enzymatic sources [85]. While some other NO donors are being investigated for their therapeutic effects (Table 1), these are in early stages and should be interpreted cautiously, as animal models do not fully replicate the complexity of human AD [86]. With regard to beetroot, betalains have been shown to suppress iNOS activity and reduce excessive NO production which may favour neuroprotective properties of nitrate-derived NO and limit neurotoxicity [48,49]. However, these studies focus on isolated mechanisms rather than considering beetroot as a whole-food matrix with potentially synergistic phytochemicals. Furthermore, although preclinical studies report antioxidant, anti-inflammatory, and neuroprotective effects of beetroot constituents, the exact compounds and mechanisms have yet to be elucidated.

In humans, research is sparse. Few studies have examined how beetroot influences the oral or gut microbiome, and even fewer have linked these changes to cognitive outcomes or markers of neurodegeneration. Moreover, the abundance of nitrate-reducing oral bacteria in individuals with AD has not been characterised, yet these microbes may influence nitrate bioavailability and, in turn, the efficacy of any dietary interventions.

Epidemiological studies have investigated dietary nitrate intake more broadly, rather than through beetroot specifically, and most focus on dementia prevention rather than disease progression. While associations exist between higher vegetable-derived nitrate intake, reduced Aβ burden, and attenuated atrophy, there is no direct evidence that these findings translate into slower AD progression. Clinical studies of beetroot report improvements in CBF, inflammation, and, in some cases, cognition, but none have been conducted in AD populations. Most prior research has used concentrated beetroot juice with single-dose, short-duration protocols, so optimal dosing schedules and effective beetroot formulations have not yet been established.

To date, only a single acute-dose study in individuals with AD has been conducted but it did not have relevant clinical endpoints. Therefore, the safety profile of long-term beetroot supplementation (especially sustained nitrate exposure) remains insufficiently characterised in AD. Individuals with AD often present with polypharmacy and comorbidities that need to be carefully considered [87].

## 5. Future Research Directions

Future preclinical studies should use whole beetroot interventions with integrative approaches that help to elucidate the mechanisms and the conditions under which beetroot-derived NO may be beneficial rather than harmful. Human studies should aim to answer several key questions, including the optimal dosage, duration, and methods of administration of beetroot. For beetroot to exert its effects, an appropriate amount of its bioactive compounds must reach the target tissues, i.e., the brain. Microencapsulation could stabilise the bioactive compounds, offer controlled release, and improve the sensorial attributes of beetroot, making it more viable as a nutraceutical [88].

Epidemiological studies would benefit from clarifying nitrate sources and mapping their associations with long-term disease trajectories, while clinical studies should adopt designs that combine metagenomic profiling, biomarkers, advanced neuroimaging, and cognitive testing within the same cohort. These studies should be explored in groups with elevated risk of AD, including individuals with mild cognitive impairment, APOE ε4 carriers, or those with vascular comorbidities. Before long-term studies can be conducted in AD populations, systematic evaluations of food–drug interactions with current AD therapies are needed [89]. Addressing these areas will clarify whether beetroot’s mechanistic promise can be translated into tangible clinical benefits, helping to clarify its role in AD management.

## 6. Conclusions

Beetroot may offer a promising avenue for further investigation in AD research because it combines vascular, antioxidant, and microbiome-modulating properties within a single whole-food source. Although the reviewed evidence points to potential benefits, particularly for neuroprotection, definitive links to clinical outcomes in AD remain unexplored. It is also important to recognise that comorbidities, medication use, and the potential for harmful elevations in NO may influence individual responses, underscoring the need for careful evaluation of safety alongside potential benefits. Further mechanistic evidence and multidomain studies are needed. Addressing these areas will establish whether beetroot can be translated into meaningful strategies for preserving brain health and slowing of cognitive decline and AD progression.

## Figures and Tables

**Table 1:** NOS-independent NO donors in animal studies.

Compounds	Neuroprotective Properties	Reference
GT 715 and GT 061 (S-nitrates)	Reversal of cognitive deficits via NO/cGMP/ERK–CREB pathways.	[37]
MN-08 (memantine with a nitrate group)	Reversal of cognitive deficits, with greater efficacy than standard memantine. Increased cerebral blood flow, reduced neuronal loss and white matter injury and activated pro-survival signalling pathways associated with neuronal resilience.	[38]
S-nitrosoglutathione	Reversal of cognitive deficits, with greater efficacy than (NOS dependent) L-Arginine. Reduced neuronal damage and amyloid β, and upregulation of brain derived neurotropic factor and Nuclear Factor Erythroid 2-Related Factor-2 antioxidant signalling pathways	[39,40]

## Data Availability

No new data were created or analyzed in this study.

## Abbreviations

The following abbreviations are used in this manuscript:

Aβ	Amyloid-β
ACC	Anterior cingulate cortex
AChE	Acetylcholinesterase
AD	Alzheimer’s disease
APOE	Apolipoprotein E
BH4	Tetrahydrobiopterin
CBF	Cerebral blood flow
cGMP	Cyclic guanosine monophosphate
DLPFC	Dorsolateral prefrontal cortex
eNOS	Endothelial nitric oxide synthases
FAD	Flavin adenine dinucleotide
IL-6	Interleukin-6
iNOS	Inducible nitric oxide synthases
LPS	Lipopolysaccharide
NADPH	Nicotinamide adenine dinucleotide phosphate
NHANES	National health and nutrition examination survey
nNOS	Neuronal nitric oxide synthases
NO	Nitric oxide
PC12	Rat adrenal pheochromocytoma cell line
ROS	Reactive oxygen species
RNS	Reactive nitrogen species
SCFA	Short-chain fatty acid
TNF-α	Tumour necrosis factor-alpha
